# Supplementary material for: Role of Leu72Met of GHRL and Gln223Arg of LEPR Variants on Food Intake, Subjective Appetite, and Hunger-Satiety Hormones
Source: Nutrients. 2022 May 18;14(10):2100. doi: 10.3390/nu14102100 (PMC9144565; doi:10.3390/nu14102100)
Supplement: Supplementary file 1 [file nutrients-14-02100-s001.zip › nutrients-1714213-supplementary.pdf]

Supplementary Materials

Table S1. Dietary intake according to Leu72Met of *GHRL* and Gln223Arg of *LEPR*.

Dietary intake	Leu/Leu (n=120)	Leu/Met + Met/Met (n=12)	p-value	Gln/Gln (n=37)	Gln/Arg + Arg/Arg (n=95)	p-value
Kilocalories (kcal)	2011.2 ± 738.3	2315.4 ± 777.6	0.180	2193.3 ± 797.7	1980.9 ± 723.9	0.191
Carbohydrates (%)	47.5± 8.5	48.3 ± 10.3	0.808	47.9± 10.6	47.4 ± 7.8	0.744
Carbohydrates (g)	238.2 ± 97.0	272.9 ± 86.3	0.153	261.7 ± 104.5	233.5 ± 93.2	0.091
Protein (%)	18.3 ± 4.1	19.2 ± 7.4	0.738	18.0 ± 4.2	18.4 ± 4.5	0.673
Protein (g)	90.5 ± 37.2	112.2 ± 56.7	0.359	100.7 ± 49.7	88.8 ± 34.4	0.567
Fat, total (%)	37.1± 7.9	35.4± 6.0	0.732	36.2± 9.3	37.3± 7.0	0.200
Fat, total (g)	84.4 ± 41.2	92.9 ± 40.4	0.528	90.4 ± 50.1	83.4 ± 37.1	0.913
Dietary cholesterol (mg)	312.2 ± 172.4	321.5± 164.9	0.786	320.7 ± 181.7	312.9 ± 167.4	0.992
SFA (g)	25.8 ± 13.9	34.1 ± 20.2	0.158	29.3 ± 19.3	25.5 ± 12.4	0.712
MUFA (g)	31.5 ± 18.4	34.0 ± 15.2	0.393	32.4 ± 21.3	31.6 ± 16.9	0.696
PUFA (g)	16.3 ± 9.1	21.9 ± 18.4	0.508	16.7 ± 10.3	17.0 ± 10.4	0.632
Sodium (mg)	2464.9 ± 1523.8	2139.1 ± 818.6	0.678	2324.2 ± 1018.2	2493.4 ± 1632.9	0.795
Dietary fiber, total (g)	26.4 ± 11.6	32.8 ± 17.2	0.219	28.9 ± 14.2	26.2 ± 11.5	0.256
Sugar, total (g)	83.3 ± 54.8	105.2 ± 42.8	0.021	102.1 ± 62.0	78.5 ± 49.8	0.033
Caffeine (mg)	34.5 ± 54.0	16.9± 19.3	0.705	32.3 ± 55.9	33.2 ± 51.0	0.527
Alcohol (g)	1.6 ± 4.3	2.5 ± 4.9	0.452	1.6 ± 4.2	1.7 ± 4.5	0.802
Food groups servings						
Bread/starch without added sugar	7.8 ± 4.1	8.3 ± 3.2	0.465	7.9 ± 4.8	7.9 ± 3.6	0.366
Bread/starch with added sugar	2.1 ± 2.2	3.5 ± 3.4	0.022	2.7 ± 2.4	1.9 ± 2.3	0.044
Fat	5.3 ± 4.4	6.0 ± 4.1	0.460	4.7 ± 3.6	5.7 ± 4.6	0.077
Fruit	1.9 ± 1.6	2.9 ± 2.2	0.036	2.3 ± 2.4	1.9 ± 1.3	0.876
Vegetables	1.8 ± 1.4	1.7 ± 1.5	0.777	1.8 ± 1.6	1.8 ± 1.3	0.725
Meat-lean	2.3 ± 2.4	2.5 ± 2.8	0.649	2.9 ± 3.1	2.0 ± 2.0	0.254
Meat-high fat	0.5 ± 0.7	0.4 ± 0.6	0.755	0.4 ± 0.7	0.5 ± 0.6	0.972
Meat-medium fat	2.2 ± 1.9	4.3 ± 5.6	0.307	2.2 ± 1.5	2.4 ± 2.8	0.825
Meat very lean	0.7 ± 1.5	0.8 ± 1.4	0.948	1.0 ± 3.0	0.6 ± 1.0	0.562
Milk-Whole	0.5 ± 0.6	0.5 ± 0.7	0.451	0.5 ± 0.7	0.5 ± 0.5	0.932
Milk -Skim	0.1 ± 0.4	0.7 ± 0.2	0.513	0.1 ± 0.3	0.1 ± 0.4	0.754
Others						

Dinner time one day before intervention (hrs)	21:25±1:05	21:00±2:06	0.568	21:23±1:24	21:23±1:09	0.995

Data are shown as mean ± SD, *p*-value < 0.05 was considered statistically significant.
SFA: saturated fatty acids, MUFA: monounsaturated fatty acids PUFA: polyunsaturated fatty acid.

Table S2. Dietary intake dinner before intervention according to Leu72Met of *GHRL* and Gln223Arg of *LEPR*.

**Variable**	**Leu/Leu n=120**	**Leu/Met + Met/Met n=12**	**p-value**	**Gln/Gln n=37**	**Gln/Arg + Arg/Arg n=95**	**p-value**
Kilocalories from dinner one day before intervention (kcal)	472.9 ± 311.1	332.8 ± 163.9	0.189	558.4 ± 386.2	387.0 ± 189.3	0.037
Carbohydrates from dinner one day before intervention (%)	53.4 ± 19.0	54.2 ± 21.0	0.903	50.4 ± 21.0	55.8 ± 17.9	0.230
Protein from dinner one day before intervention (%)	18.2 ± 9.1	17.8 ± 14.2	0.913	18.3 ± 9.3	17.7 ± 9.9	0.808
Fat, total from dinner one day before intervention (%)	30.9 ± 14.6	30.1 ± 13.8	0.868	33.6 ± 15.7	29.1 ± 13.9	0.198
Available carbohydrates from dinner one day before intervention (g)	1.1 ± 3.5	1.5 ± 4.1	0.661	1.1 ± 3.6	0.9 ± 3.2	0.821
Alcohol from dinner one day before intervention (g)	0.0 ± 0.2	0.0 ± 0.0	0.661	0.0 ± 0.0	0.0 ± 0.2	0.372

Data are shown as mean ± SD, *p*-value < 0.05 was considered statistically significant.
